# Prioritizing Conservation Areas for the Hyacinth Macaw ( *Anodorhynchus hyacinthinus* ) in Brazil From Low‐Coverage Genomic Data

**DOI:** 10.1111/eva.70039

**Published:** 2024-11-18

**Authors:** Sibelle Torres Vilaça, Jeronymo Dalapicolla, Renata Soares, Neiva Maria Robaldo Guedes, Cristina Y. Miyaki, Alexandre Aleixo

**Affiliations:** ^1^ Instituto Tecnológico Vale Belém Pará Brazil; ^2^ Departamento de Sistemática e Ecologia Universidade Federal da Paraíba João Pessoa Paraíba Brazil; ^3^ Instituto de Biociências Universidade de São Paulo São Paulo São Paulo Brazil; ^4^ Instituto Arara Azul Campo Grande Mato Grosso do Sul Brazil; ^5^ Programa de Pós‐graduação em Meio Ambiente e Desenvolvimento Regional Universidade para o Desenvolvimento do Estado e da Região do Pantanal Campo Grande Mato Grosso do Sul Brazil

**Keywords:** Arara‐azul‐grande, conservation genetics, genomic monitoring, isolation by environment, parrots, Psittacidae

## Abstract

Estimates of current genetic diversity and population connectivity are especially important for endangered species that are subject to illegal harvesting and trafficking. Genetic monitoring can also ensure that management units are sustaining viable populations, while estimating genetic structure and population dynamics can influence genetic rescue efforts and reintroduction from captive breeding and confiscated animals. The Hyacinth Macaw ( *Anodorhynchus hyacinthinus* ) is a charismatic endangered species with a fragmented (allopatric) distribution. Using low coverage genomes, we aimed to investigate the dynamics across the remaining three large disjunct populations of Hyacinth Macaws in Brazil to inform conservation strategies. We obtained low coverage DNA data for 54 individuals from seven sampling sites. Our results showed that Hyacinth Macaws have four genetically structured clusters with relatively high levels of diversity. The Pantanal biome had two genetically distinct populations, with no obvious physical barriers that might explain this differentiation. We detected signs of gene flow between populations, with some geographical regions being more connected than others. Estimates of effective population size in the past million years of the species' evolutionary history showed a decline trend with the lowest *Ne* in all populations reached within the last few thousand years. Our findings suggest that populations from the Pantanal biome are key to connecting sites across its distribution, and maintaining the integrity of this habitat is important for protecting the species. Given the genetic structure found, we also highlight the need of conserving all wild populations to ensure the protection of the species' evolutionary potential.

## 1 Introduction

Landscape alterations and habitat fragmentation are changing species dynamics. These modifications can isolate populations, leading to higher rates of inbreeding, and genetic drift, which ultimately decrease genetic diversity and long‐term evolutionary potential (Frankham 2010). The use of genetic monitoring for endangered species is ever more needed to guarantee that conservation units are sustaining viable populations, while estimates of gene flow among these units are crucial to understanding current and past dynamics that can influence genetic rescue efforts. Studies using whole genome sequences have increased the resolution to detect fine genetic differentiation and recent gene flow dynamics (Ewart et al. 2020). These parameter estimates are especially important for endangered species that are subject to illegal harvesting and trafficking since reintroduction of confiscated animals or those from captive breeding programs can benefit from knowing the source population for potential targets of reintroduction to minimize extinction risk (Aliaga‐Samanez et al. 2023).

**FIGURE 1:**
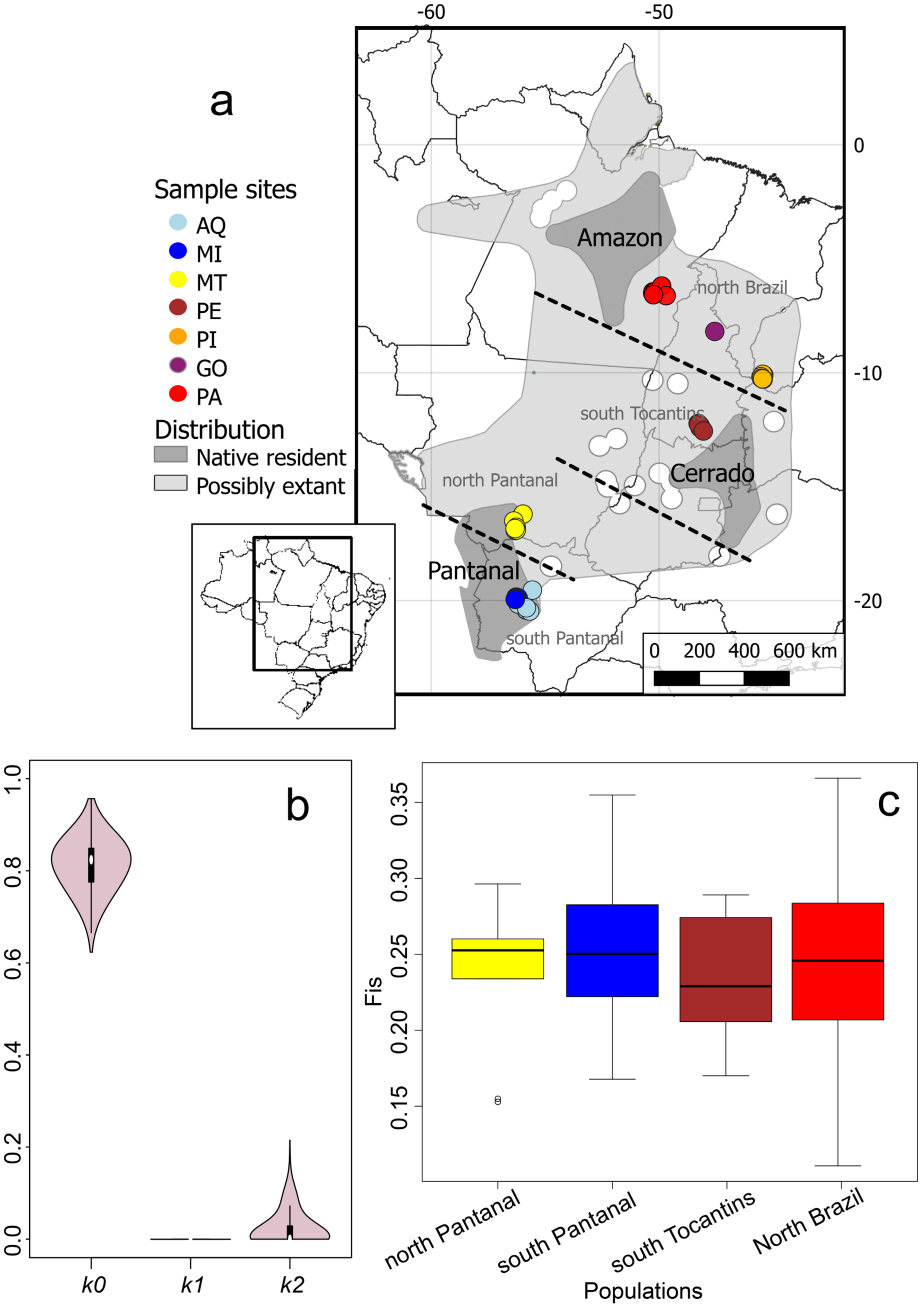
(a) Map depicting sampling sites. Areas where the Hyacinth Macaws is possibly extinct are shown as hollow circles within the distribution (light gray), with dark gray polygons indicating current main strongholds in the species' distribution (Birdlife International 2024). Distribution map kindly provided by BirdLife International (2023). Black lines denote state border lines, while dashed lines represent potential barriers to gene flow. (b) Pairwise relatedness estimates for all analyzed samples. (c) Inbreeding (Fis) estimates per genetically isolated grous. Colors refer to sampling sites. AQ = Aquidauana; MI = Miranda; MT = Barão de Melgaço; PA = Canaã dos Carajás; PE = Peixes, PI = São Gonçalo do Gurguéia.

The Hyacinth Macaw ( *Anodorhynchus hyacinthinus* ) is a charismatic species with a high risk of extinction (Guedes, Bianchi, and Barros 2008). It is classified as vulnerable by the IUCN Red List (IUCN 2016) and as endangered by the Brazilian Red List (ICMBio 2018). This is the largest flying parrot with up to 1 m of wingspan, characterized by social behavior and generally found in monogamic pairs up to groups of 100 s of individuals (Guedes et al. 2022; Scherer‐Neto, Guedes, and Toledo 2019). They also exhibit strong fidelity to nesting and feeding areas (i.e., philopatry) (Guedes and Harper 1995). Mating pairs have been reported to return to the same breeding sites, demonstrating a phylopatric behavior combined with widespread habitat use (Faria et al. 2008; Guedes et al. 2021). The Hyacinth Macaw occurs mainly in Brazil in the Pantanal wetlands (Mato Grosso and Mato Grosso do Sul states), and parts of the Cerrado (Goiás, Tocantins, western Bahia and southern Maranhão and Piauí states), and the Amazon centered in southern Pará, with marginal individuals recorded near the Pará‐Amazonas border and in southern Amapá (Devenish et al. 2021; Guedes, Bianchi, and Barros 2008) (Figure 1). Marginal records are also found near the Bolivia‐Brazil, and Paraguay‐Brazil borders (Devenish et al. 2021). Censuses estimate that the number of wild individuals varies between 4300 and 6500 (BirdLife International 2016; Faria et al. 2008; Guedes, Bianchi, and Barros 2008). Numbers of Hyacinth Macaws have been greatly reduced due to habitat destruction and illegal nest extraction, with estimates that ~10,000 individuals were illegally captured for pet trade during the 1980s (BirdLife International 2016). However, conservation efforts have reversed declining trends at least locally in the Pantanal (Guedes et al. 2021; ICMBio 2018; Scherer‐Neto, Guedes, and Toledo 2019; Vicente and Guedes 2021). There are currently three main largely disjunct areas of Hyacinth Macaw occurrences (Collar, Boesman, and Sharpe 2020) corresponding to regions of higher individual density in the Pantanal, Amazon, and Cerrado biomes (dark gray polygons in Figure 1a), which might be connected to some extent (Devenish et al. 2021). Therefore, estimating fine‐scale structure and connectiveness among the extant population areas of occurrence is important for potentially improving management strategies.

Previous genetic studies of Hyacinth Macaws using microsatellite and mitochondrial DNA markers have found overall low genetic diversity compared to other macaw species (Faria et al. 2008) with three highly genetically structured populations based on nuclear microsatellites and mitochondrial sequences (North/Northeast, North Pantanal, and South Pantanal) (de Almeida et al. 2019; Faria et al. 2008; Presti et al. 2015). The evidence of two differentiated populations within Pantanal was unexpected, given that free movement of individuals was initially assumed within this region due to the lack of a clear physical barrier separating them (de Almeida et al. 2019; Presti et al. 2015). Putative explanations for this pattern included the absence of registered animals in intermediate regions possibly due to lack of critical nesting and/or feeding habitat (Presti et al. 2015; Antas, pers. comm.). Similarly, mitochondrial DNA data showed signs of population structure between south and north Pantanal in agreement with known philopatric behavior of both males and females (de Almeida et al. 2019). The use of higher‐density molecular markers, like re‐sequenced genomes, might improve the resolution of genetic structure within the extant populations and help elucidate the extent of gene flow between them.

The main objective of our study was to characterize the population dynamics across the range of Hyacinth Macaws in Brazil by increasing the resolution of markers using low coverage whole genomes. We aimed to: (i) confirm if genomic data recover similar population genetic structure and number of management units obtained from previous studies using microsatellites; (ii) calculate the connectivity across populations and infer if any population is isolated, which could increase the species' risk of extinction; and (iii) estimate the population dynamics and levels of genetic diversity to infer the risk of extinction for each population based on effective population size and inbreeding analyses.

## 2 Material and Methods

### 2.1 Sampling

We obtained 54 samples from wild Hyacinth Macaws from seven locations across a significant part of the species' range (Figure 1a; Table S1). We collected blood samples from the wing vein of nestlings that were immediately returned to their nests after the procedure. Samples were collected under animal ethics permit (#188/2013‐IB 2013.1.941.41). Samples were obtained from two sites in the Pantanal wetlands: from Mato Grosso do Sul state (Aquidauana AQ; *n* = 10; and Miranda MI; *n* = 10), and from Mato Grosso state (Barão de Melgaço, MT; *n* = 10). The other sites were distributed as follows: São Gonçalo do Gurguéia in Piauí state (PI; *n* = 6); Canaã dos Carajás in Pará state (PA; *n* = 8); plus Peixes (PE; *n* = 9) and Goiantins (GO; *n* = 1) in Tocantins state. DNA was extracted using the DNeasy Blood & Tissue kit (Qiagen) and quantified using the dsDNA HS assay in Qubit 3.0 (Life Technologies). We used 25 ng of each sample's genomic DNA for library preparation with the SureSelectQXT kit (Agilent Technologies). Libraries were sequenced in our in‐house Illumina NextSeq550 using a high 300‐cycle high‐output kit.

### 2.2 Genotype Likelihood Calculations

We used FastQC v0.11.9 (Andrews 2010) to assess read quality for each individual. Adaptors and low‐quality bases (*Q* < 20) were removed with Trimmomatic v0.32 (Bolger, Lohse, and Usadel 2014). We mapped all reads to a scaffold‐level Hyacinth Macaw genome (Hains et al. 2020) (NCBI accession number: GCA_009936445.1, N50 = 3.2 Mb) using BWA mem v0.7.17 (Li and Durbin 2009). Optical read duplicates were marked with Picard v2.17.8 (Broad Institute 2009) and unmapped reads removed with samtools v.1.15.1 (Danecek et al. 2011). We used ANGSD (Korneliussen, Albrechtsen, and Nielsen 2014) to calculate genotype likelihoods given the read coverage of our samples (~2×) using the GATK caller (‐GL 2). We used similar filters as Dalapicolla et al. (2024) and kept biallelic SNPs (‐skipTriallelic) in reads of good quality (‐remove_bads 1; ‐uniqueOnly 1; ‐only_proper_pairs 1; −baq 1 ‐C 50 ‐trim 0), with significant mapping (‐minMapQ 20; ‐mapQ_pval 0.05) and base call quality (‐min*Q* 20; ‐*q*score_*p*val 0.05); with a significant *p*‐value for SNP detection (‐SNP_*p*val 1e‐6); minor allele frequency (‐minMaf) of 0.05; kept SNPs in at least 50% of individuals (‐minInd 27); removed strand bias (‐sb_*p*val 0.01), edge bias (‐edge_*p*val 0.05), and sites with excess heterozygosity (‐hetbias_*p*val 0.05) (Fumagalli et al. 2014; Lou et al. 2021). We additionally removed SNPs with mean coverage < 0.8× (‐setMinDepth) and with mean coverage across individuals that exceeded the median value of coverage depth for all SNPs +2× its standard deviation (‐setMaxDepth 50). (Fox et al. 2019). To prune linked sites, we used ngsLD (Fox et al. 2019) to calculate linkage disequilibrium for a pair of SNPs, and the LD decay rate was estimated using PopLDdecay v3.42 (Zhang et al. 2019). Based on the LD decay rate graphs, SNPs were filtered with a minimum distance (‐‐max_dist) of 200 bp and a minimum weight (‐‐min_weight) of 0.5 (Dalapicolla et al. 2024; Fox et al. 2019; Galla et al. 2018). Unlinked SNPs were kept for further analysis using the option ‐‐sites in ANGSD. As an outgroup, we used reads from the Scarlet Macaw *Ara macao* (GenBank accession number SRR25548345) (Aardema, Schmidt, and Amato 2023). Reads were mapped and filtered following the same pipeline as for Hyacinth Macaw samples.

### 2.3 Population Structure, Diversity, and Demography

To estimate population structure, we performed a Principal Component Analysis (PCA) using PCAngsd and an admixture analysis using ngsAdmix (Meisner and Albrechtsen 2018). For the admixture, we tested the number of clusters (K) from 1 to 10 with 10 replicates and calculated the best K using Evanno's test (Evanno, Regnaut, and Goudet 2005) in the online tool CLUMPAK (Kopelman et al. 2015). We also calculated pairwise F_ST_ values using ANGSD to estimate population differentiation among sampling sites. For the F_ST_ estimates, variant filters similar to the described above were used (‐dosaf 1 ‐gl 1 ‐remove_bads 1 ‐uniqueOnly 1 ‐only_proper_pairs 1 ‐baq 1 ‐C 50 ‐trim 0 ‐minMapQ 20 ‐minQ 20). Calculations were done separately for each sampling site and genetically structured population (the latter based on PCA and ngsAdmix results) to avoid biases due to population structure.

To estimate the genetic diversity for extant populations, we estimated observed genome‐wide nucleotide diversity (Θπ), Watterson's Theta (Θ_W_), and inbreeding (F_IS_) for each sample locality. The F_IS_ was calculated using ngsF with a min_epsilon = 0.000001. The summary statistics of heterozygosity, nucleotide diversity (Θπ), and Watterson's Theta (Θ_W_) were estimated from each genetically isolated population's unfolded Site Frequency Spectrum (SFS) following the ANGD's manual (http://www.popgen.dk/angsd/index.php/Thetas,Tajima,Neutrality_tests). The Θπ and Θ_W_ were calculated for non‐overlapping windows of 10,000 bp, and the values obtained were divided by 10,000 to account for the window length. The site allele frequency likelihood (SAF) used for the SFS estimates followed the same filters as for the F_ST_. To confirm that all individuals were unrelated, which may interfere in the detection of population structure and skew diversity estimates (Manichaikul et al. 2010), we estimated potential relatedness among our samples, using ngsRelate (Korneliussen and Moltke 2015) from genotype likelihoods. We used parameter settings similar to previous studies (Margaryan et al. 2020; Vilaça et al. 2023), with maximum likelihood estimates (−m 1), a log‐likelihood difference lower than 10e^−6^ between two consecutive EM‐steps (−t 1e‐06) as a stopping criterion for the EM algorithm, and a maximum number of steps of 10,000 (−i 10,000). We evaluated pairwise relatedness using the coefficients for non‐inbred relatives *k*_0_, *k*_1_, and *k*_2_. Kinship was inferred based on expected values: monozygous twins (*k*_0_ = 0, *k*_1_ = 0, *k*_2_ = 1), parent‐offspring (*k*_0_ = 0, *k*_1_ = 1, *k*_2_ = 0), full‐sibs (*k*_0_ = 0.25, *k*_1_ = 0.5, *k*_2_ = 0.5); half‐sibs, uncle‐nephew, or grandparent‐grandchild (*k*_0_ = 0.5, *k*_1_ = 0.5, *k*_2_ = 0).

To infer the effects of potential inbreeding on individuals of Hyacinth Macaw, we estimated the proportion of the genome in runs of homozygosity (F_ROH_). Long regions of homozygosity throughout the genome (> 1 Mb) are used to detect recent inbreeding (Foote et al. 2021), and longer ROHs denote inbreeding in the immediately preceding generations (McQuillan et al. 2008). We used Rohan (Renaud et al. 2019) which estimates heterozygosity (as Waterson's Θ) in low coverage samples. We tested two parameter combinations of rohmu and window lengths: the default combination rohmu = 1e‐05, window length = 1 Mb; and rohmu = 2e0‐4, window length = 500 kb. This last combination used a rohmu closer to the estimated Watterson's theta value and allowed for smaller ROH detection given our scaffold length choice (details below). Because the reference genome used to map our reads is at a scaffold‐level, and, thus, very fragmented, and since we are interested in recent inbreeding that might be influencing the Hyacinth Macaw's long‐term survival (ROHs > 1 Mb; Foote et al. 2021), we only used scaffolds longer than 1 Mb. We avoided detecting regions in short scaffolds that are not associated with ROHs, and therefore used only scaffolds > 1 Mb in this analysis, avoiding including scaffolds that might not reflect true ROHs in our draft‐level reference genome. We extracted 12 scaffolds > 1 Mb totaling 567.1 Mb (corresponding to 51% genome) using bedtools and estimated the ROH regions.

We estimated gene flow between groups with the *D*‐statistics (ABBA‐BABA) to calculate conflicting patterns between ancestral (“A” alleles) and derived (“B” alleles), and therefore assess the differential contributions of gene flow and incomplete lineage sorting (ILS). Excesses in the “ABBA” or “BABA” patterns produce deviation from *D* = 0, supporting gene flow. We used the command abbababa2 in ANGSD to calculate values considering multiple individuals per population. The Scarlet Macaw was set as the outgroup. Significant gene‐flow was considered if the *Z*‐score > 3 and was assessed using jackknifing (Durand et al. 2011).

The dynamics of effective population size (*Ne*) through time was estimated using Stairway Plot v2.1.1 (Liu and Fu 2020) with SNPs frequency spectrum (SFS) to estimate *Ne* dynamics in the past 150,000 years. Estimates of long‐term *Ne* can provide a context to current levels of genetic diversity (Wilder et al. 2023), while also providing an indication on which biogeographical and climatic patterns might have influenced changes in past *Ne* dynamics (Dalapicolla et al. 2024). Given our interest in both ancient and recent *Ne* dynamics, we used the unfolded SFS to avoid wider confidence intervals due to loss of information (Liu and Fu 2020) calculated using realSFS in ANGSD. We set the parameter length (*L*) as the number of SNPs in each population (range: 43,603 to 105,869 SNPs) multiplied by the linkage disequilibrium distance (200 bp). The substitution rate was set as 4.6 × 10^−9^ (Smeds, Qvarnström, and Ellegren 2016) and generation time as 11.29 years (Bird et al. 2020).

### 2.4 Landscape Genetics

To estimate possible barriers in the landscape that could influence gene flow, we used the Estimated Effective Migration Surfaces (EEMS) (Petkova, Novembre, and Stephens 2015). The EEMS uses an isolation‐by‐resistance approach to estimate the genetic dissimilarity between geo‐referenced individuals under a stepping‐stone model to assess observed migration rates from expected values (Giorello, D'Elía, and Lessa 2021). By estimating the gene flow across adjacent demes and interpolating the results as surface maps, it is possible to visualize barriers and corridors for movement across the investigated landscape. We simulated 500 demes and 10 independent runs, each for 5 million iterations, 50% burn‐in and sampling every 1000 iterations. To check convergence, we used Tracer v1.7 (Rambaut et al. 2018). Parameters were adjusted so runs had the recommended acceptance proportion of 20%–30% (Petkova, Novembre, and Stephens 2015).

Genetic connectivity between sites was estimated using a population graph framework through the conditional genetic distance (cGD) statistic (Dyer 2015; Dyer and Nason 2004). The cGD is defined as the length of the shortest path through the topology of connections among the analyzed populations (Dyer 2015) and is calculated from the genetic covariance among sampling sites. We used the popgraph package (Dyer 2015; Dyer and Nason 2004) and graph4lg (Savary et al. 2021) in R (R Core Team 2017). These methods consider the principle of conditional independence in which populations with similar covariant allelic frequencies, when accounting for the covariance in all populations, are shown as connected in the graph. The genetic distance between sites with at least 4 individuals was calculated using the function popgraph with 0.05 significance adjusted by the correction of Benjamini and Hochberg (1995). For each site, we calculated *betweenness*, which corresponds to the sum of the shortest pathways within the graph length that connects two sites. To estimate connectivity across sites, we used the *weight* as an indicator of the connection strength between two sites (Dyer 2015). Results were visualized as a population graph in which nodes represent sampled sites, and edges represent genetic connections among the sites.

We tested whether our dataset follows the isolation‐by‐distance (IBD) model by performing a Mantel test (Mantel 1967) at the individual level using the mantel.rtest function with 10,000 permutations in the “ade4” R package (Dray and Dufour 2007). We calculated a geographical (Euclidean) distance matrix based on distances between individuals using the distm function in the package “geosphere” (Hijmans 2019). For genetic distance, we estimated PCAdistance between individuals applying the eigenvalues found in PCangsd analyses and the function “ecodist” 2.0.7 (Goslee and Urban 2007) based on the Mahalanobis distance (Shirk, Landguth, and Cushman 2017). We retained 50 PCs corresponding to 95% of the variance found in the PCA. We chose a PCA‐based distance metric because it showed a good performance in landscape genetics analyses and it can be interpreted similarly to Fst used in classical population‐based Mantel tests (Shirk, Landguth, and Cushman 2017).

## 3 Results

### 3.1 Population Structure, Dynamics, and Genetic Diversity

We obtained 357,086,224 paired reads for 54 samples (Figure 1a) with an average coverage of 2.31× (2.05 to 3.20×) after quality control and mapping (Table S1). Linkage disequilibrium results indicated a low *r* ^2^ even in nearby SNPs (Figure S1). Our relatedness estimates showed for all pairwise comparisons a *k*_0_ > 0.6, *k*_1_ = 0 and *k*_2_ < 0.2 indicating no kinship among analyzed individuals (Figure 1b); therefore, all samples were considered in our analysis. Considering the total homozygous regions along the genome (F_ROH_), we did not observe ROH regions > 1 Mb in most of our samples, and only three individuals from Aquidauana had a higher proportion (> 10%) of its genome in ROH (Figure S2 and Table S2) in one of the tested parameter combinations.

**FIGURE 2:**
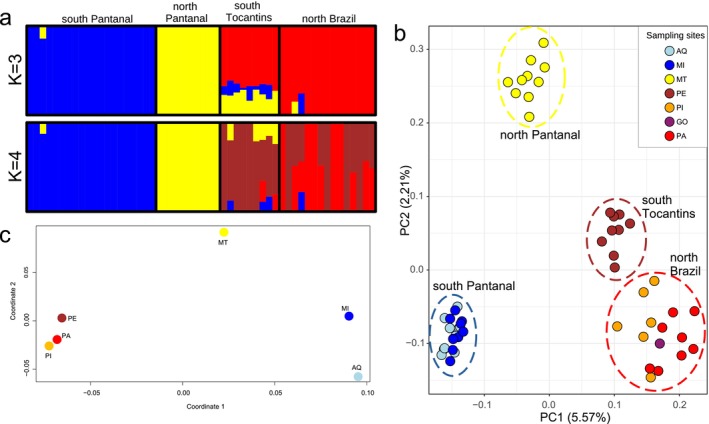
Population structure of Hyacinth Macaws. (a) Admixture plot showing the estimated ancestry proportions using NGSadmix for *K* = 3 and *K* = 4. For each *K*‐value, 10 runs were performed, and CLUMPAK was used to estimate the major mode. Each partitioned vertical bar represents an individual's proportional membership to the inferred populations. (b) Principal component analysis (PCA) from PCAngsd. (c) Multidimensional scaling (MDS) analysis of pairwise F_ST_ values calculated with ANGSD. Colors refer to sampling sites from Figure 1a. The GO population was excluded from F_ST_ calculations as it has only one sample. AQ = Aquidauana; MI = Miranda; MT = Barão de Melgaço; PA = Canaã dos Carajás; PE = Peixes, PI = São Gonçalo do Gurguéia.

Admixture analysis reflected clustering subdivisions observed in previous studies, with no finer signals of population structure. Considering all samples (Figure 2a), we obtained a preferred *K* = 3 (Figure S2) and retrieved the clusters of south Pantanal (AQ, MI), north Pantanal (MT), and northern Brazil (PA, PE, PI, GO), with a further subdivision at *K* = 4 within northern Brazil in south Tocantins (PE) and north Brazil (PA, PI, GO). A similar structure was found in the Principal Component Analysis (PCA) (Figure 2b). In the PCA, the PC1 separated the three large populations similarly to the admixture analysis. Within the south Pantanal group, we observed that individuals from two sampling sites largely overlap. When considering the PC2, the south Tocantins group is divided from the rest of the north Brazil individuals in agreement with the results from ngsAdmix.

Pairwise F_ST_ statistics showed low differentiation between south Tocantins and north Brazil (Fst = 0.08), with higher values amongst all other populations (F_ST_ ~ 0.12 to 0.16), including between north and south Pantanal (Table S3). Pairwise F_ST_ values within clusters, as defined by Structure and PCA, were smaller than between clusters (F_ST AQ × MI_ = 0.06; F_ST PA × PI_ = 0.09) (Table S4). Thus, the structure pattern from F_ST_ values was similar to Structure and PCA results (Figure 2c). Estimates of individual‐levels of inbreeding (F_IS_) were similar for most isolated populations (F_IS_ ~ 0.25; Figure 1c). However, the north Pantanal population had a higher mean inbreeding value (F_IS_ = 0.27) than other sites. Heterozygosity was higher in south Pantanal, while north Brazil had higher values of Θπ and Θ_W_ (Table S5).

Gene flow estimates from D‐statistics (ABBA‐BABA) showed that gene flow (either historical or current) is prevalent within and between clusters. The highest values of *D* involved gene flow between north and south Pantanal (Table 1). When considering the sampling sites separately, gene flow was higher within south Pantanal (AQ and MI), indicating a high migration rate between these two sites (Table S6). We also observed gene flow between south Tocantins and the Pantanal (Table 1 and Table S6). Our overall results indicate that gene flow is common but can vary between specific sites within clusters. For example, we observe significant D values between MI (south Pantanal) and PA (north Brazil) (e.g., D_((MT,MI) PA)_ = 0.004, Z‐score = 4.58; Tables S6 and S7), but not between AQ (south Pantanal) and PA. This demonstrates differential connectivity within sites of south Pantanal and north Brazil populations, which might indicate that gene flow is location dependent. In general, the D‐stats results show that some sampling sites (e.g., MT) might serve as a “hub” for migration routes (see also Landscape genetic section), despite the lack of widespread gene flow among all sites and existing population structure.

**TABLE 1:** D‐statistics analysis between genetically differentiated clusters.

*D*	*Z*	*p*	nABBA	nBABA	H1	H2	H3
−0.099	−118.876	0	180,920.8	221,017.9	**north Pantanal**	north Brazil	**South Pantanal**
−0.052	−59.053	0	182,499.4	202,541	**north Pantanal**	South Tocantins	**south Pantanal**
0.103	126.243	0	221,017.9	179,697.5	North Brazil	**south Pantanal**	**north Pantanal**
−0.051	−58.071	0	183,050.7	202,541	**South Pantanal**	South Tocantins	**North Pantanal**
0.055	71.372	0	206,673.8	185,045.9	north Brazil	**South Tocantins**	**North Pantanal**
−0.052	−63.875	0	186,319.3	206,673.8	**north Pantanal**	North Brazil	**south Tocantins**
0.051	66.908	0	207,610.2	187,636.8	north Brazil	**south Tocantins**	**south Pantanal**
0.049	64.861	0	207,610.2	187,852.1	North Brazil	**south Pantanal**	**south Tocantins**
0.003	4.406	0.000011	180,920.8	179,697.5	North Pantanal	**south Pantanal**	**North Brazil**
0.003	4.262	0.00002	186,319.3	185,045.9	north Pantanal	**south Tocantins**	**north Brazil**

*Notes:* Only significant tests (*Z*‐score > |3|, *p* < 0.05) are shown. Gene flow between clusters, as defined in Figure 2, are denoted in bold. Excess of ABBA (supporting introgression between H2 and H3 over incomplete lineage sorting) are shown by positive values, while excess of BABA (supporting introgression between H1 and H3) are shown by negative values. The Scarlet Macaw was used as an outgroup.

**FIGURE 3:**
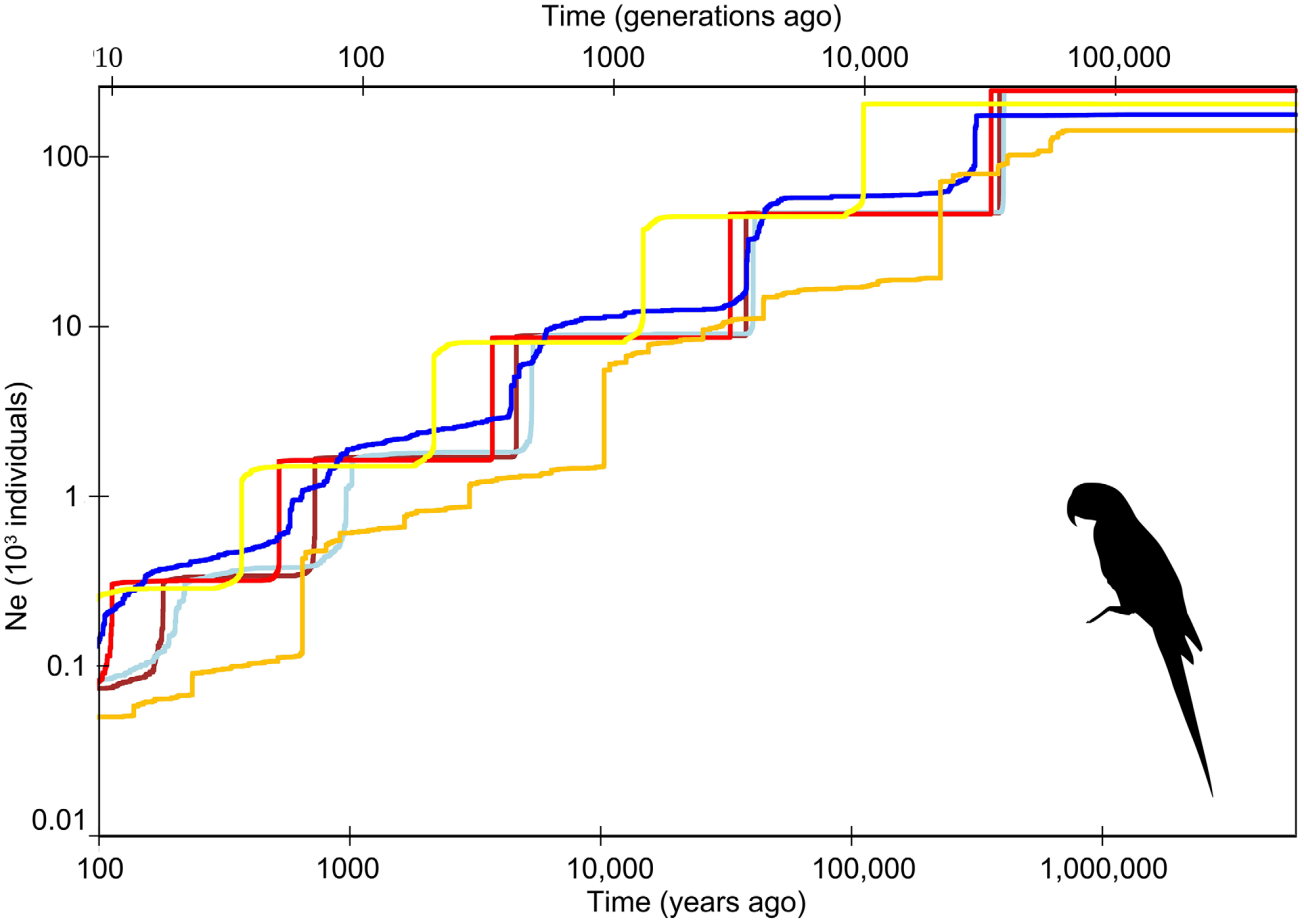
Past demographic history for each sampled population of Hyacinth Macaws. The GO population was excluded as it has only one sample. The stairway plot shows the historical effective population size (*Ne*, *y* axis) over the past generations (upper *x*‐axis) and years (lower *x* axis). Colors represent population structure from Figure 1.

Demographic history analysis using the Stairway Plots showed effective population size (*Ne*) fluctuations throughout the evolutionary history of the populations (Figure 3). The overall pattern was a decrease in *Ne* over time in all populations with largely similar estimates of *Ne* throughout time, with an accentuated decline in the past few hundred years (Figure 3).

### 3.2 Landscape Genetics

**FIGURE 4:**
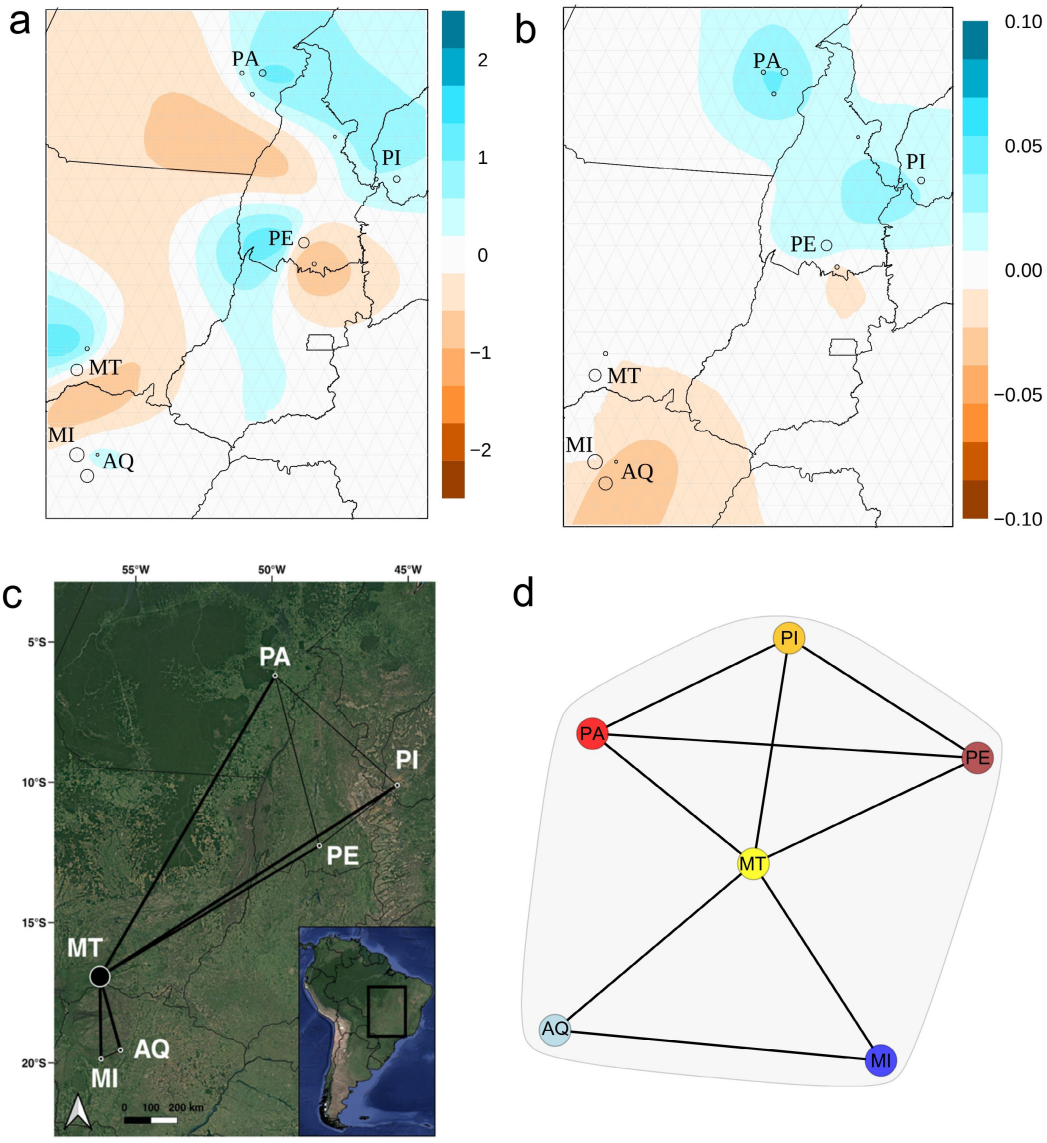
Landscape genetics analysis. (a) Estimated migration rate surface produced by EEMS showing posterior mean migration rates (m) between sample sites in logarithmic scale. Blue shades indicate higher migration rates than expected, while red shades indicate reduced connectivity. The convergence plot is shown in Figure S2. (b) Posterior average genetic diversity (*q*) in logarithmic scale. Blue shades indicate higher diversity, while red shades indicate more homogenous areas than expected. (c) Popgraph network analysis for *Anodorhynchus hyacinthinus* populations. Circle sizes reflect the levels of genetic variation within populations (betweenness). Lines weight represents the genetic variation due to connecting node (*weight*), reflecting higher gene flow among sites. (d) Schematic representation of main connectivity from popgraph results. AQ = Aquidauana; MI = Miranda; MT = Barão de Melgaço; PA = Canaã dos Carajás; PE = Peixes; and PI = São Gonçalo do Gurguéia.

Graphs of population connectivity showed that the MT sampling site (north Pantanal) is an important connectivity site given the high values of *betweenness* and *weight* (Figure 4c,d and Table S8). The migration surface produced by EEMS showed two potential barriers to gene flow, one between south and north Pantanal and another running roughly in a northwest‐southwest direction across the state of Tocantins (Figure 4a). However, the delimitation for the Tocantins barrier may be imprecise due to lack of sampling on both sides of this hypothetical barrier (Petkova, Novembre, and Stephens 2016), thus we do not consider it as a potential barrier. We identified a connectivity corridor in north Brazil, in agreement with the lack of structure within the north Brazil population based on Structure and PCA results (Figure 4a). In addition to effective migration, the effective genetic diversity within each deme showed that the Pantanal as a whole harbors lower diversity (Figure 4b), in agreement with summary statistics (Table S8). Conversely, samples within the north Brazil cluster are more genetically diverse than expected (Figure 4b), in agreement with high gene flow estimates within this group and with Pantanal sites (Figures 2a and 3). Our Mantel tests showed a marginally significant result (*r* = 0.074; *p* = 0.042), indicating Hyacinth macaws follow an IBD model (Figure S5).

## 4 Discussion

Here, using data from low‐coverage genomes, we show that imperiled Hyacinth Macaws have a strong population structure and relatively high levels of genetic diversity. We also show signs of gene flow between populations, with some geographical regions being more connected than others. Population dynamics analysis showed a decreasing trend in effective population size throughout the species' range and along its evolutionary history that has been accentuated more recently. Based on our results, we discuss implications for its conservation.

### 4.1 Population Structure and Connectivity

Hyacinth Macaws currently have three main population strongholds located in the Pantanal, southeastern Amazonia, and Central Brazil (BirdLife International 2016). While it is thought to have once been one continuous distribution, habitat loss combined with illegal captures for trade have led to a decrease in habitat and local extinction of geographically intermediate populations (Machado, Drummond, and Paglia 2008). Previous studies using microsatellite markers and mitochondrial DNA sequences showed genetic structure in Hyacinth Macaws, with four main genetically structured populations recognized: north Brazil, south Tocantins, south Pantanal, and north Pantanal (de Almeida et al. 2019; Presti et al. 2015). Our results are largely in agreement with those obtained in these previous studies, although we observed greater differentiation between the so‐called north and northeast populations than Presti et al. (2015) and de Almeida et al. (2019), corresponding, respectively, to our north Brazil (PA, PI, and GO) and south Tocantins populations (Figure 2). Our results from ngsAdmix (Figure 2a) also show less admixture with more markedly structured populations at *K* = 3 in Pantanal compared to Presti et al. (2015), even though we found comparable values of F_ST_, inbreeding, and heterozygosity (Tables S3 and S4).

Hyacinth Macaws are subject to illegal harvesting due to their high value in the trade market. Fortunately, conservation efforts have contributed to increase the number of individuals, bringing the species from a categorization of Endangered to Vulnerable (BirdLife International 2016). Most recovery efforts are concentrated in the Pantanal, where the largest census numbers is located with ca. 5000 of its 6500 estimated individuals (BirdLife International 2016; Scherer‐Neto, Guedes, and Toledo 2019). Interestingly, our results have confirmed with whole‐genome data that the Pantanal is a unique region for the Hyacinth Macaw, with two highly genetically structured populations found across a relatively small geographic area (Figure 2a,b). This substructure between north/south Pantanal has not yet been observed in other animals (Presti et al. 2015). Although no obvious physical barriers can be evoked to explain this differentiation, intrinsic biological features of the species and/or past refugia might render this structure pattern unique to Hyacinth Macaws (see below).

Population structure not associated with physical barriers has been observed in other macaws in Central and South America, and other parrot species in different continents (Ewart et al. 2020). The critically endangered Red‐fronted Macaw ( *Ara rubrogenys* ; Blanco et al. 2021) has a structure pattern largely explained by complex social factors reinforcing philopatry‐related genetic distance. However, in Scarlet Macaws, the two subspecies *Ara macao* *cyanoptera* and *A. m. macao* showed almost complete isolation due to a large volcanic mountain chain in Costa Rica based on mitogenomes (Schmidt, Aardema, and Amato 2020), with a lack of structure within subspecies in analyzed individuals from Mexico to Brazil. Furthermore, microsatellites showed fine‐scale structure in Scarlet Macaws on the Pacific coast of Costa Rica, with strong differentiation over an 80 km distance possibly associated with historical barriers or elevation (Monge et al. 2016). Isolation‐by‐elevation across populations separated by 20 km was also found in Scarlet Macaws from the Peruvian Amazon based on nuclear markers (Olah et al. 2017). Compared to other Neotropical psittacines, mitochondrial DNA patterns recovered for the Hyacinth Macaw showed that female philopatry played a limited role in population differentiation (Faria et al. 2008; Schmidt, Aardema, and Amato 2020). Similar to the Red‐fronted Macaw, the Hyacinth Macaw also has philopatric behavior, and combined with effective limited dispersal and a gregarious social organization, genetic structuring might arise independent of physical barriers. Even if Hyacinth Macaws can fly long distances, their movement distance averages 20 km with an average home range of 10,481 ha (Antas et al. 2010; Presti et al. 2015). Therefore, we hypothesize that, like other macaws, the genetic clustering of Pantanal in north and south populations might be derived from complex social behaviors inherent to their biology.

Another hypothesis for the north/south structure within Pantanal might be associated with suitable habitats associated with subregions. The Pantanal biome has 10–11 regions, depending on the classification of different vegetation types, soil, relief, and flooding regimens (da Silva and de Moura Abdon 1998; da Silva et al. 2000). Each of our sample sites in Pantanal (MI, AQ, and MT) comes from a different subregion. Although the two subregions in south Pantanal (MI and AQ) are composed of predominantly *cerradão* tall woodland, the less predominant phytophysiognomy varies in these two areas (da Silva et al. 2000). The third sampling site, in north Pantanal, Barão de Melgaço (MT) is covered mostly by *cerrado* savannas. Consequently, even though the Pantanal regions do not have strong physical barriers separating them (Presti et al. 2015), the vegetation subregions might lead to isolation by habitat. Hyacinth Macaws are strongly associated with manduvi trees ( *Sterculia apetala* ) for nesting cavities and have the Acuri palm (*Attalea phalerata*) as a main food source in the Pantanal (Guedes, Bianchi, and Barros 2008; Oliveira et al. 2021). Distribution models showed that predicted suitable habitats for both plants vary along the biome, with a large region of high flooding intensity and lower suitability for both palm species running across the middle and western portions of the central Pantanal (Oliveira et al. 2021). Interestingly, *S. apelata* was modeled at a much higher density in southern than northern Pantanal, whereas the opposite was true for *A. phalerata* , which could indicate that these keystone resources might be utilized at different frequencies by Hyacinth Macaws in southern and northern Pantanal, potentially driving ecological and even cultural divergences between these two genetically distinct populations (Oliveira et al. 2021). The environmental heterogeneity associated with philopatry and social behaviors may help explain the geographical clustering between north and south populations. Since past changes of habitat can also influence the current distribution of genetic diversity, further studies associating changes of these habitats during the Pleistocene can elucidate the timing and consequences of past environmental changes to the current population structure observed in Hyacinth Macaws from Pantanal.

Individual dispersal associated with gene flow is an important mechanism to avoid inbreeding depression (Pike, Cornwallis, and Griffin 2021; Pusey and Wolf 1996). Considering the landscape genetics analysis over all sites, our results suggest that the north Pantanal connects the Pantanal region to the remaining north Brazil and south Tocantins populations (Figure 4c,d), as also demonstrated by the significant levels of gene flow from D‐statistics (Figure 3). Our popgraph provides evidence that MT (north Pantanal) is a key site allowing genetic connectivity throughout the species' distribution outside the Pantanal, with some gene flow also present with the north Brazil/Tocantins populations (Figure 4c,d). This connectivity also agrees with our ngsAdmix results, as gene flow from Pantanal to north Brazil/Tocantins is observed (Figure 2a). It also corroborates diversity estimates (Θπ, Θ_W_; Table S3), since north Brazil sites (PA, PI) show higher diversity than other locations possibly due to gene flow. A recent temporal modeling study showed that the extent of occurrence of the Hyacinth Macaw in the Amazon has increased in recent decades, suggesting that deforestation could be playing a role in driving this expansion, while it remained relatively stable in the Pantanal and Cerrado biomes during the same period (Devenish et al. 2021). This study also hinted that Hyacinth Macaw's wide‐ranging habits when searching for resources (mainly palms, which were found to be species' best predictors of occurrence) could lead to long‐distance seasonal movements (Devenish et al. 2021), which has not yet been confirmed by empirical evidence. Our whole‐genome data is consistent with long‐range connectivity between the northern Pantanal and the remaining populations sampled outside this biome (Figure 4). These patterns support the hypothesis of long‐range movements across the Hyacinth Macaw's distribution centered in the northern Pantanal populations, which could act as a main source of individuals establishing new populations in the Amazon and Cerrado biomes. Furthermore, our genomic data does not rule out a more complex scenario, with individuals of some populations breeding locally in the Amazon and Cerrado (potentially driving observed genetic differentiation in these populations), while others could still exhibit philopatric behavior while occupying a large area by flying long distances, to return to breeding areas in the northern Pantanal. The genetic connectivity verified amongst the non‐Pantanal sites (PA, PE, and PI) nevertheless confirms that dispersal and perhaps regular movements also occur within the Amazon and between the Cerrado and Amazon biomes. Under an evolutionary perspective, the strong genetic structure among groups (Figure 2a) combined with landscape estimates of recent connectivity (Figure 4) show that gene flow might be due to recent processes. Further sampling and discovery of new Amazon and Cerrado areas where the species is present (which currently are thought to have about 500 and 1000 individuals, respectively; Faria et al. 2008) will provide a better resolution of the connectivity between them and the more numerous Pantanal area (ca. 5000 individuals; Devenish et al. 2021; Faria et al. 2008). In summary, our results confirm the crucial importance of the Pantanal region to the overall long‐term survival of the species.

### 4.2 Genetic Diversity

Hyacinth Macaws are classified as vulnerable by the IUCN Red List (BirdLife International 2016) and as endangered by the Brazilian Red List (ICMBio 2018; MMA et al. 2018), but do not show concerning low levels of genetic diversity as previously thought based on two microsatellite loci and mtDNA (Faria et al. 2008). Our diversity statistics (Table S3) show that while south Pantanal has higher heterozygosity, north Brazil has higher estimates of Θπ and Θ_W_. The combination of these results indicates that while south Pantanal has a within population relative high diversity, the differences between individuals (i.e., Θπ) is higher in north Brazil, possibly due to gene flow mediated by migrants from other populations. The interplay of these two factors demonstrates the importance of understanding the species' dynamics and structure. Our results failed to detect signs of recent inbreeding from long (> 1 Mb) ROH regions in most of the samples that were analyzed. Admittedly, our low coverage data combined with the reference genome available at the scaffold‐level, did not allow for detection of ROHs in its entirety. Although we are estimating ROHs in 51% of the genome, widespread signs of ROHs would appear in the longer scaffolds analyzed. The F_IS_ estimate (a proxy for inbreeding) is not as high when compared to other endangered birds like the Brown eared pheasant ( *Crossoptilon mantchuricum* ) (Wang et al. 2021) with a similar number of individuals (BirdLife International 2016). However, an isolated population with inferred inbreeding coefficient of 0.1 (F_IS_ > 0.1) requires management intervention (Ralls et al. 2018). The Hyacinth Macaw, although not an isolated population, has an inferred F_IS_ > 0.2, which warrants genetic monitoring of its diversity.

Our estimates of past population dynamics showed a long‐term *Ne* decrease consistent across all populations sampled over at least the last 100,000 years (Figure 3). Contrary to previous studies that failed to detect past bottlenecks with microsatellite data (Presti et al. 2015), we show that all sampled populations had a similar declining trend over the previous interglacial, last glacial, and Holocene periods (Figure 4). The pattern of long‐term *Ne* decrease, compounded by more recent reductions related to hunting and trafficking, which might have started with the earliest signs of human presence in South America (Pansani et al. 2023), might have driven the more recent *Ne* estimates to the lowest values in the Hyacinth Macaws evolutionary history (see Bergman et al. 2023). However, gradual population declines are associated with purging of strongly deleterious mutations and an accumulation of mildly deleterious variants due to less efficient selection in small populations (Grossen et al. 2020; Robinson et al. 2018, 2023). Future genomic datasets with higher coverage depths, associated with a chromosome‐level reference genome, will allow for a robust testing of the impacts of the observed long‐term demographic decline documented herein in the mutation load of the Hyacinth Macaw.

### 4.3 Implications for Conservation

Our study provides key insights into conservation genetics and management strategies for Hyacinth Macaws. Knowledge of genetic variability is an important parameter to allow species' long‐term survival. While many endangered species show decrease in genetic diversity, a direct relationship between IUCN Red List status and genetic diversity is not straightforward (Garner, Hoban, and Luikart 2020; Schmidt et al. 2023). Conservation efforts have significantly increased the number of Hyacinth Macaw individuals in the Pantanal (Scherer‐Neto, Guedes, and Toledo 2019) and apparently across most of its range, particularly in the Amazon (Devenish et al. 2021), and we encourage these should continue to ensure that no further population declines affect the species' long‐term survival. Even though past studies have suggested that biological traits, such as monogamy and low recruitment rate, contribute to a higher extinction probability in Hyacinth Macaws (Oliveira et al. 2021), our study shows that from a genetic standpoint, the future survival of this charismatic species might not be as dire as previously thought (Faria et al. 2008).

Our results also demonstrate that there are at least four genetic clusters among the remaining populations of the Hyacinth Macaw, distributed as follows: north Pantanal, south Cerrado (corresponding to our Tocantins cluster), and north Cerrado + Amazonia (corresponding to our Pará, northern Tocantins, and Piauí cluster) (Figure 2). Thus, we recommend these populations should be managed separately. Increasing efforts of additional sampling in other areas of the species' presence based on species modeled distribution areas might lead to possible undiscovered populations (*sensu* Devenish et al. 2021). Genomic data might refine further population structure, especially on adaptive variation related to their strong dependence of trees as food source and nesting (as described above). Our study also contributed to show the putative impacts of deforestation and habitat fragmentation on dispersion. Hyacinth Macaws are illegally harvested for its feathers and market value of live individuals. Seizing of adults and chicks in Brazil is frequent, and knowledge of putative population structure is important for reintroduction of seized individuals and also uncovering trafficking routes of these birds (Presti et al. 2015). Therefore, the knowledge of fine‐scale genetic clustering can aid in identifying the source of seized birds, preventing the release of individuals in a different population of origin. While our study demonstrates that low‐coverage data can give insights into the structure and dynamics of endangered populations, whole genomic data can also contribute to help law enforcement reintroduce seized individuals back to nature. Another important step will be the assembly of a chromosome‐level genome for Hyacinth Macaws, an effort already underway by our group, which, associated with higher‐coverage data will allow further estimates and monitoring of genetic erosion in Hyacinth Macaws.

## Conflicts of Interest

The authors declare no conflicts of interest.

## Supporting information


Figure S1.

Table S1.

## Data Availability

Raw Illumina sequences have been deposited at NCBI’s Sequence Read Archive (SRA) under BioProject PRJNA1181145.
