# Supplementary material for: Prioritizing Conservation Areas for the Hyacinth Macaw ( Anodorhynchus hyacinthinus ) in Brazil From Low‐Coverage Genomic Data
Source: Evol Appl. 2024 Nov 18;17(11):e70039. doi: 10.1111/eva.70039 (PMC11573696; doi:10.1111/eva.70039)
Supplement: Supplementary file 1 — Figure S1. [file EVA-17-e70039-s001.docx]

**Supplementary Figures**


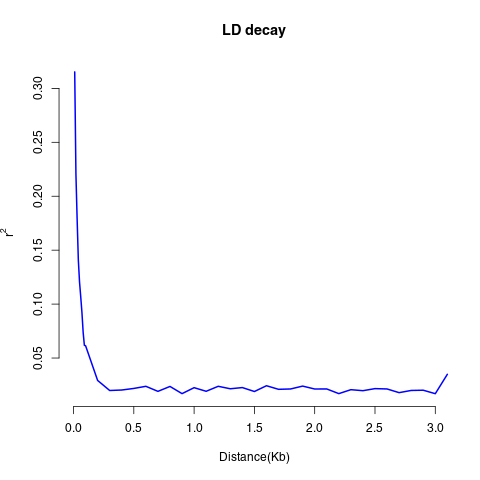

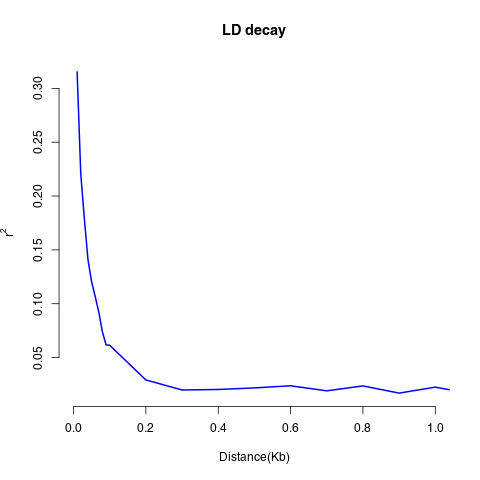

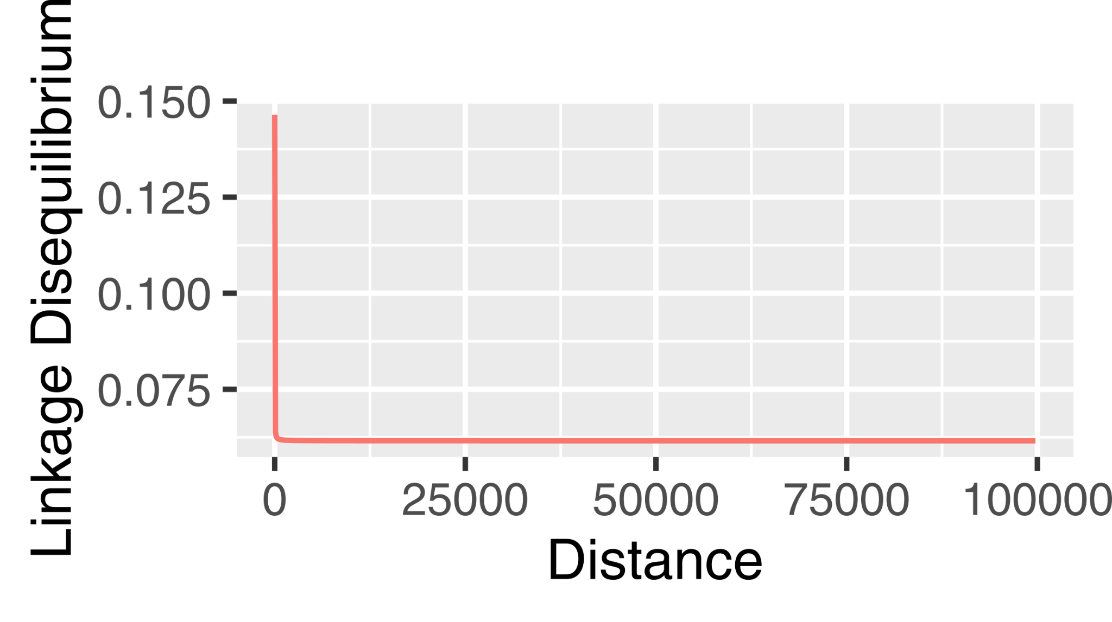


A

B

C

**Figure S1** –Linkage disequilibrium (LD) decay rate calculated for each pair of SNPs using the maximum distance of 1 Kb (A), 3 Kb (B) in PopLDdecay v3.42, and using the ngsLD v1.1.1 (C), showing low values of r2 even in short distances as 200 bp, the value used as a threshold for filtering unlinked SNPs.


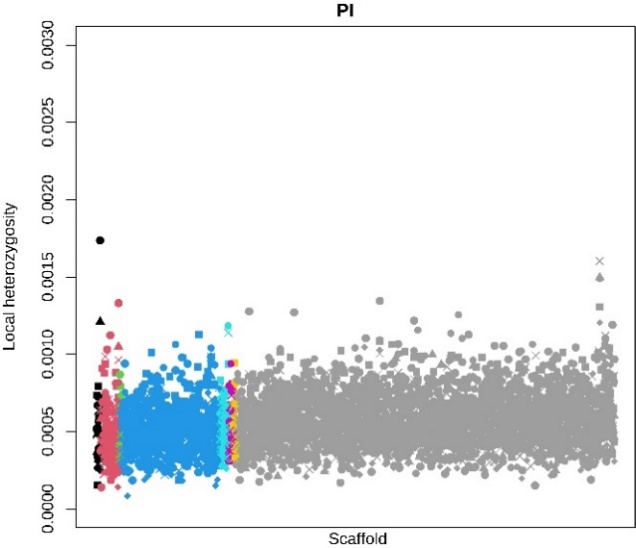

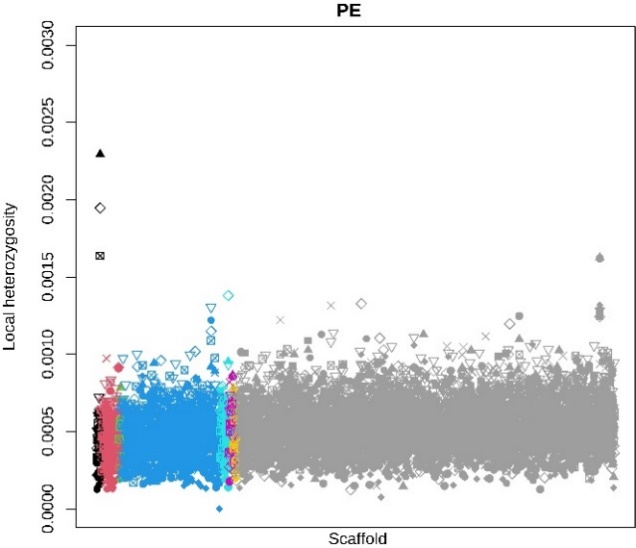

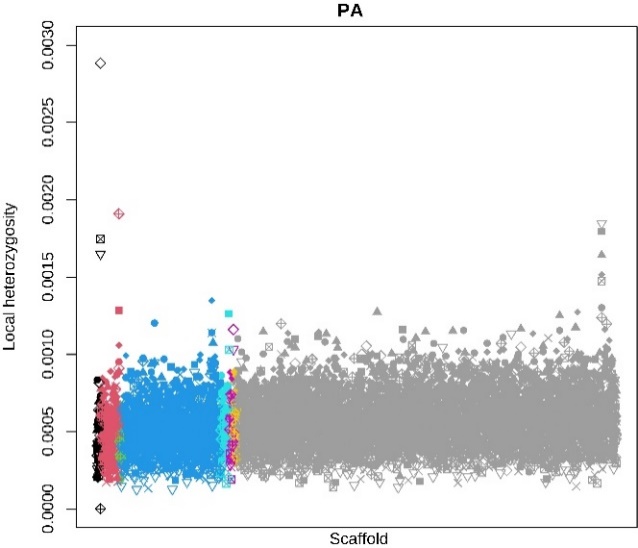

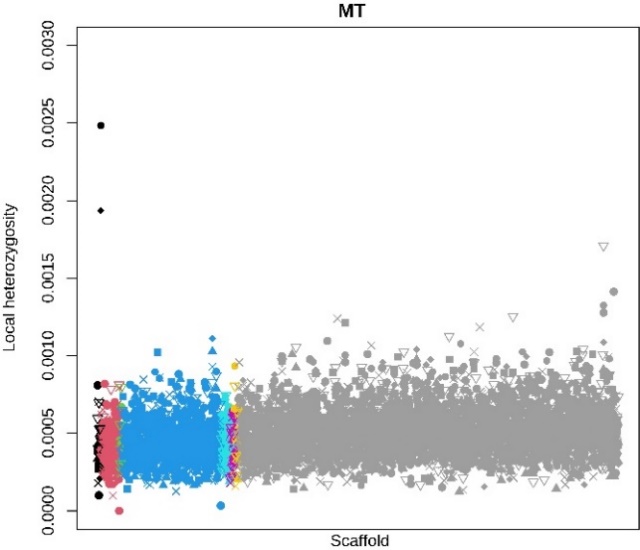

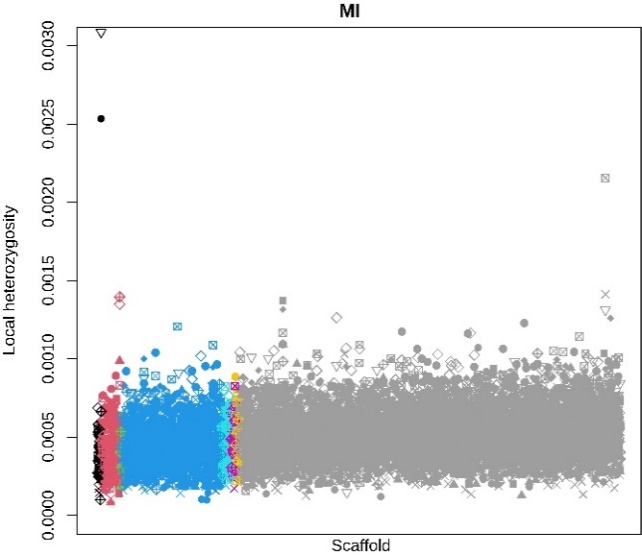

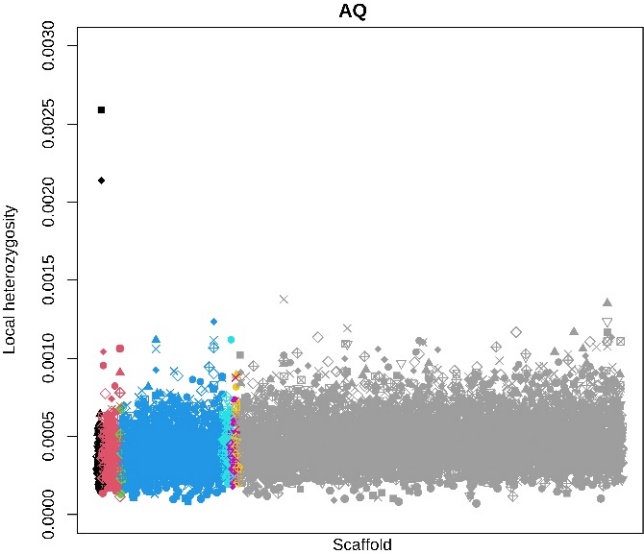
**Figure S2** – Local heterozygosity plots per population (AQ, MI, MT, PA, PE, PI) showing the lack of long runs of homozygosity. Each scaffold is represented by one color, and different individuals are represented by different symbols. Only eight scaffolds are shown as an example. Each point represents a 500kb window.


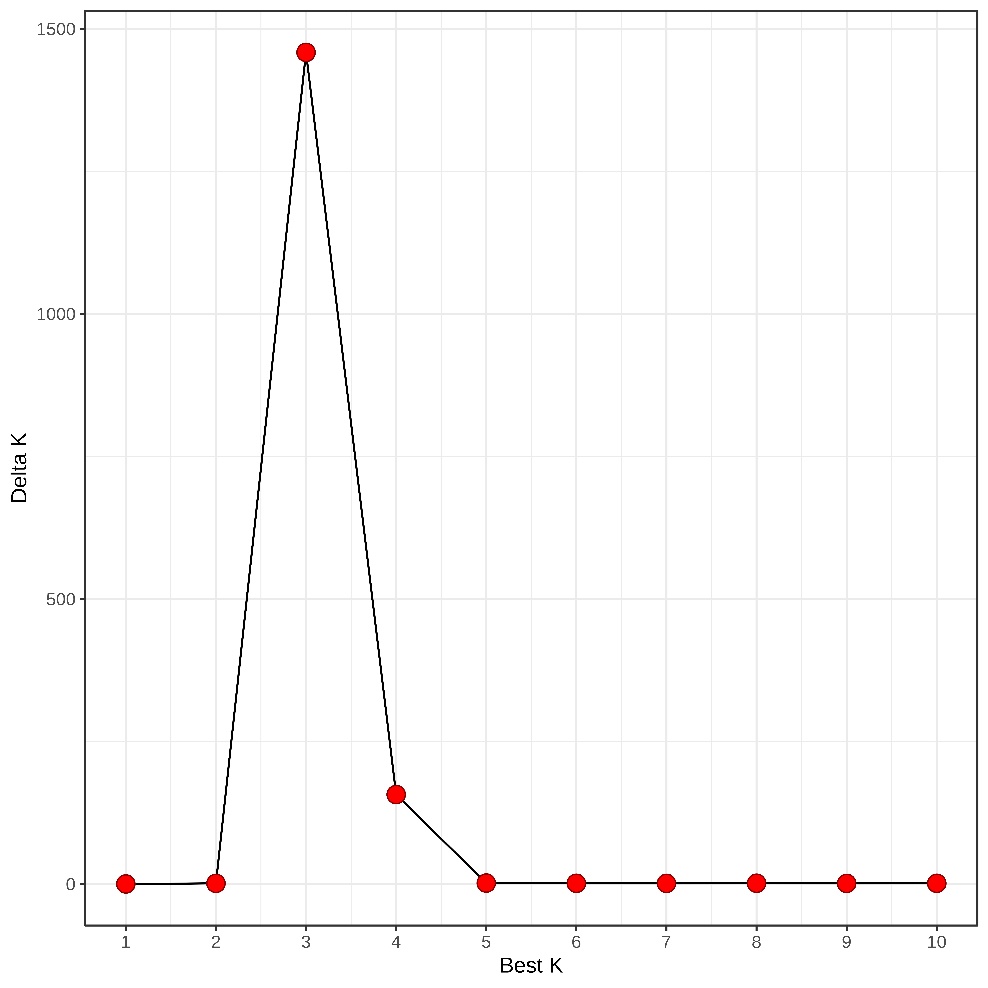
**Figure S3** – Delta K plot for ngsAdmix analysis showing the optimal K by Evanno is K = 3.


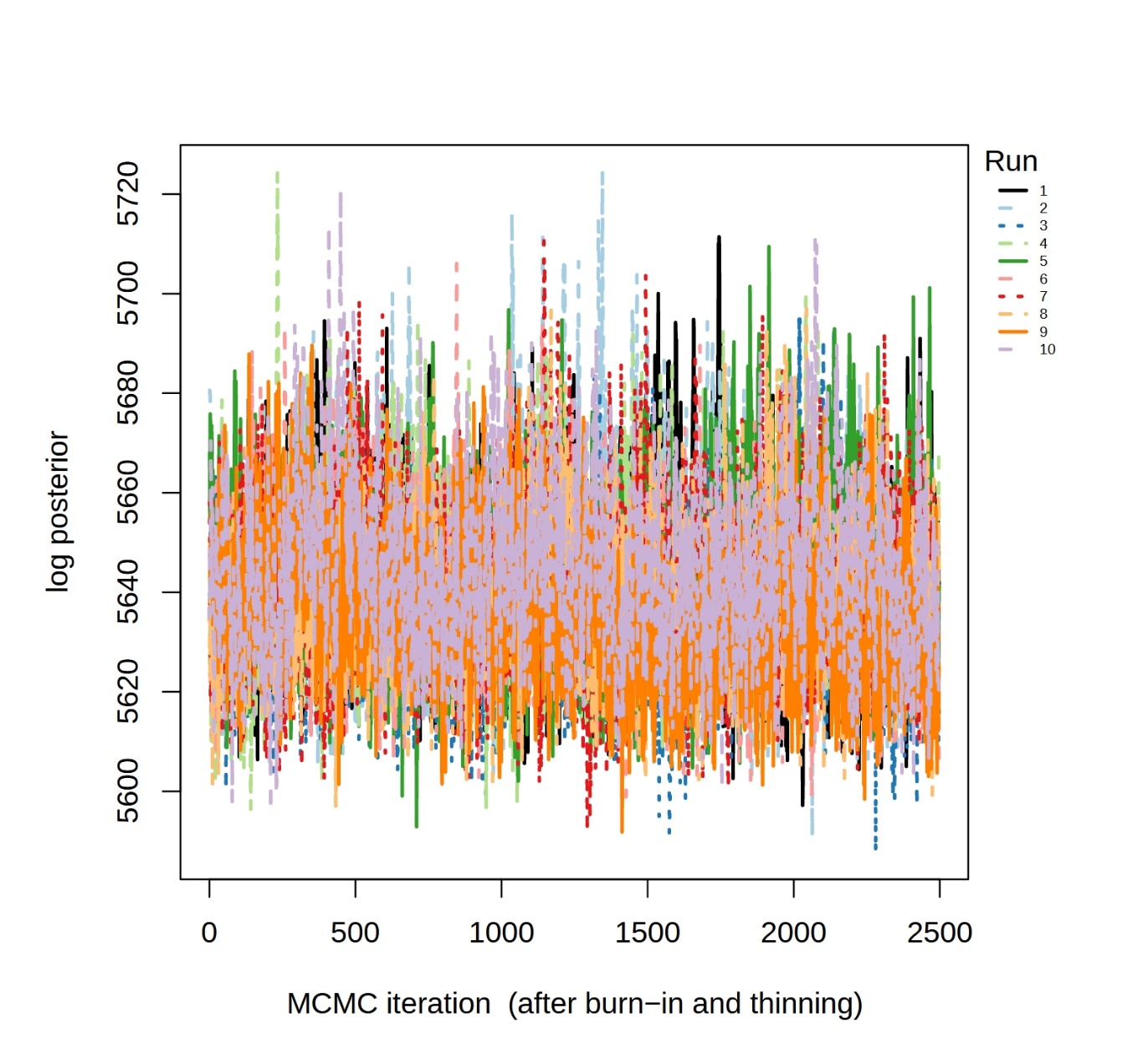


**Figure S4** – Convergence plot for EEMS analysis.


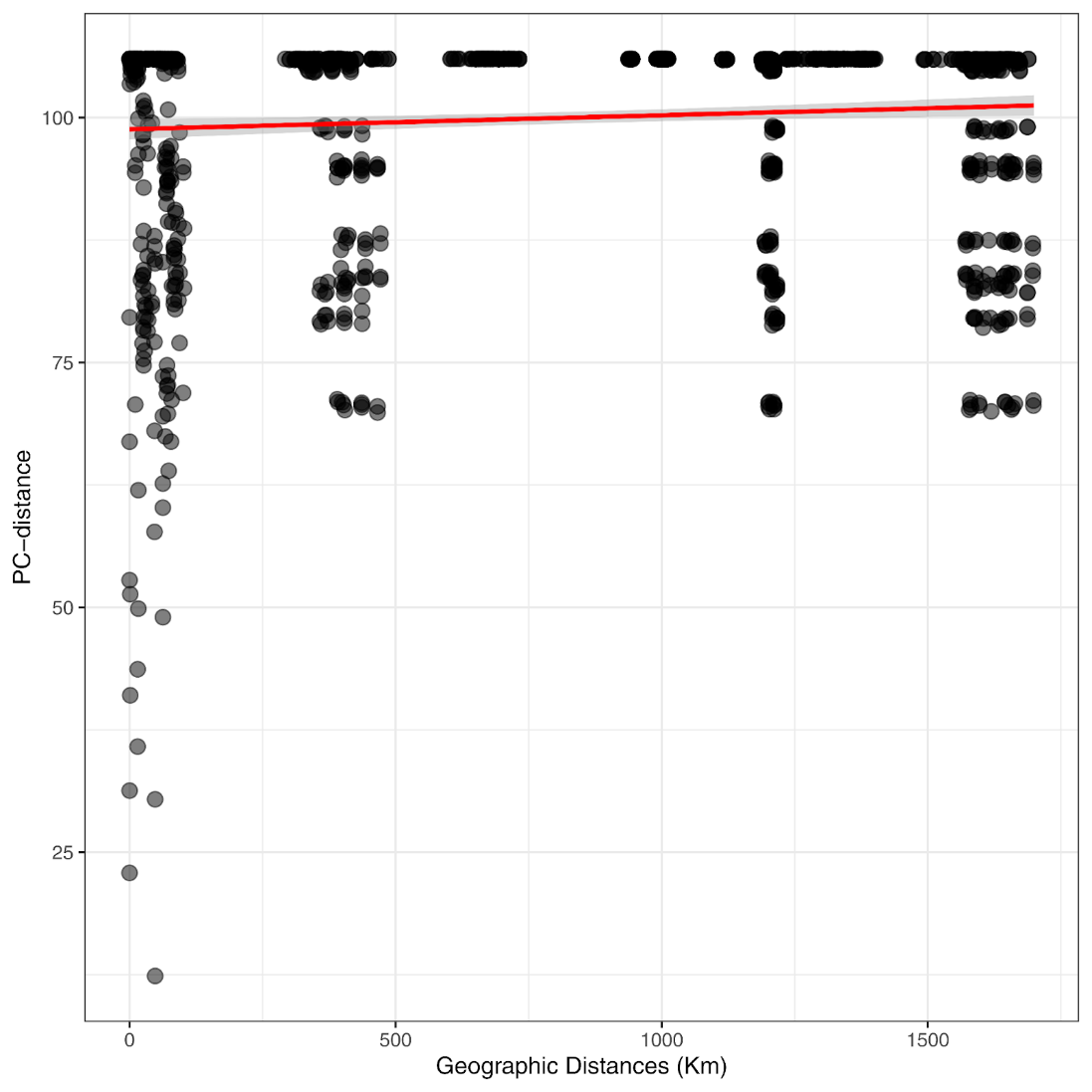


**Figure S5** – Mantel test per individual. The X-axis denotes the geographical (Euclidean) distance, while the Y-axis shows the eigenvalues found in PCangsd based on Mahalanobis distance.
